# Supplementary material for: Declining Levels of Neutralizing Antibodies to SARS-CoV-2 Omicron Variants Are Enhanced by Hybrid Immunity and Original/Omicron Bivalent Vaccination
Source: Vaccines (Basel). 2024 May 22;12(6):564. doi: 10.3390/vaccines12060564 (PMC11209254; doi:10.3390/vaccines12060564)
Supplement: Supplementary file 1 [file vaccines-12-00564-s001.zip › vaccines-2981423-supplementary.pdf]

Demographic Characteristics	STOPCoV study	Sub-study	P-value
**Total (N)**	983	178	-
**Age, n (%)**			
30-50 years	248 (25.2%)	52 (29.2%)	0.2638
70+ years	735 (74.8%)	126 (70.8%)	
Mean (SD)	65.67 (15.30)	66.28 (15.32)	0.6231
Median (IQR)	72 (50, 75)	73 (50, 76)	<u>0.0065</u>
**Gender, n (%)**			
Female	631 (64.2%)	130 (73.0%)	<u>0.0224</u>
Male	352 (35.8%)	48 (27.0%)	
**BMI, n (%)**			
Obesity (BMI ≥ 30)			
No	746 (75.9%)	133 (74.7%)	0.7374
Yes	237 (24.1%)	45 (25.3%)	
Mean (SD)	27.1 (5.5)	27.4 (6.0)	0.4781
Median (IQR)	26.2 (23.3, 29.8)	25.8 (23.5, 30.0)	0.825
**Racial background, n (%)**			
White	872 (88.7%)	160 (89.9%)	0.6449
Other	111 (11.3%)	18 (10.1%)	
**Smoking status, n (%)**			
Never	559 (56.9%)	103 (57.9%)	0.7863
Previous	386 (39.3%)	70 (39.3%)	
Current	38 (3.9%)	5 (2.8%)	
**Comorbidities, n (%)**			
Diabetes			
No	877 (89.2%)	157 (88.2%)	0.6899
Yes	106 (10.8%)	21 (11.8%)	
Cardiovascular Disease			
No	626 (63.7%)	110 (61.8%)	0.631
Yes	357 (36.3%)	68 (38.2%)	
Respiratory diseases			
No	868 (88.3%)	165 (92.7%)	0.0849
Yes	115 (11.7%)	13 (7.3%)	
Cancer			
No	824 (83.8%)	137 (77.0%)	<u>0.0258</u>
Yes	159 (16.2%)	41 (23.0%)	
Transplant			
No	941 (95.7%)	170 (95.5%)	0.8933
Yes	42 (4.3%)	8 (4.5%)	

Table S1: Comparison of the demographics of the sub-study participants to those of the main STOPCoV study, SD-, standard deviation, n- number, IQR- interquartile range, BMI –body mass index, Bonferroni family-wise error threshold of 0.005.

	Hybrid immunity	Vaccine Only immunity	Total	P-value
**Age group 30-50 years**	**27**	**25**	**52**	**-**
**Wuhan: Neutralizing Antibody, n (%)**				
Non-detectable (= 0)	-	-	-	-
detectable (> 0)	27 (100%)	25 (100%)	52 (100%)	-
Log10 1/ID50				
Mean (SD)	3.92 (1.05)	3.78 (0.62)	3.85 (0.86)	0.5492
Median (IQR)	4.14 (3.17, 4.47)	3.81 (3.36, 4.24)	3.99 (3.26, 4.35)	0.5156
**Omicron BA.5: Neutralizing Antibody, n (%)**				
Non-detectable (= 0)	1 (3.7%)	3 (12.0%)	4 (7.7%)	0.262
detectable (> 0)	26 (96.3%)	22 (88.0%)	48 (92.3%)	
Log10 1/ID50				
Mean (SD)	3.41 (0.57)	2.69 (0.55)	3.08 (0.66)	<.0001
Median (IQR)	3.44 (2.98, 3.87)	2.64 (2.47, 2.97)	3.01 (2.64, 3.59)	<.0001
**Omicron XBB.1.5: Neutralizing Antibody, n (%)**				
Non-detectable (= 0)	4 (14.8%)	14 (56.0%)	18 (34.6%)	0.0018
detectable (> 0)	23 (85.2%)	11 (44.0%)	34 (65.4%)	
Log10 1/ID50				
Mean (SD)	2.60 (0.42)	2.06 (0.30)	2.42 (0.46)	0.0006
Median (IQR)	2.62 (2.22, 2.82)	1.96 (1.79, 2.48)	2.49 (2.02, 2.77)	0.0007
**Omicron EG.5: Neutralizing Antibody, n (%)**				
Non-detectable (= 0)	6 (22.2%)	16 (64.0%)	22 (42.3%)	0.0023
detectable (> 0)	21 (77.8%)	9 (36.0%)	30 (57.7%)	
Log10 1/ID50				
Mean (SD)	2.46 (0.41)	1.88 (0.24)	2.29 (0.45)	0.0005
Median (IQR)	2.46 (2.11, 2.64)	1.83 (1.66, 2.06)	2.22 (1.88, 2.56)	0.0005
**Age group 70+ years**	**65**	**61**	**126**	**-**
**Wuhan Neutralizing Antibody, n (%)**				
Non-detectable (= 0)	-	-	-	-
detectable (> 0)	65 (100%)	61 (100%)	126 (100%)	-
Log10 1/ID50				
Mean (SD)	4.43 (0.87)	3.80 (0.75)	4.12 (0.87)	<.0001
Median (IQR)	4.55 (3.96, 4.83)	3.74 (3.45, 4.12)	4.04 (3.63, 4.67)	<.0001
**Omicron BA.5: Neutralizing Antibody, n (%)**				
Non-detectable (= 0)	1 (1.5%)	9 (14.8%)	10 (7.9%)	0.0061
detectable (> 0)	64 (98.5%)	52 (85.2%)	116 (92.1%)	
Log10 1/ID50				
Mean (SD)	3.74 (0.51)	2.58 (0.60)	3.22 (0.79)	<.0001
Median (IQR)	3.73 (3.45, 4.05)	2.57 (2.15, 2.92)	3.24 (2.58, 3.88)	<.0001
**Omicron XBB.1.5: Neutralizing Antibody, n (%)**				
Non-detectable (= 0)	6 (9.2%)	38 (62.3%)	44 (34.9%)	<.0001
detectable (> 0)	59 (90.8%)	23 (37.7%)	82 (65.1%)	
Log10 1/ID50				
Mean (SD)	2.87 (0.47)	2.11 (0.58)	2.66 (0.60)	<.0001
Median (IQR)	2.88 (2.61, 3.26)	1.89 (1.71, 2.41)	2.77 (2.23, 3.14)	<.0001
**Omicron EG.5: Neutralizing Antibody, n (%)**				
Non-detectable (= 0)	10 (15.4%)	45 (73.8%)	55 (43.7%)	<.0001
detectable (> 0)	55 (84.6%)	16 (26.2%)	71 (56.3%)	
Log10 1/ID50				
Mean (SD)	2.69 (0.50)	2.13 (0.46)	2.56 (0.54)	0.0002
Median (IQR)	2.64 (2.37, 3.04)	1.93 (1.83, 2.44)	2.61 (2.12, 2.98)	0.0003

Table S2: The proportion of participants with detectable neutralizing antibody and the titers in those with detectable levels by age cohort to Wuhan and Omicron variants BA.5, XBB 1.5 and EG.5. Grouping is according to hybrid/vaccine immunity at baseline.- SD-standard deviation, IQR- interquartile range, n- number.

Variables	Logistic regression (detectable vs non-detectable)		Linear Regression (detectable)	
	Univariable (OR)	Multivariable (adOR)	Univariable (β)	Multivariable (adβ)
**Wuhan**				
Age:70+ years	-	-	0.27 (-0.01, 0.55)	0.06 (-0.25, 0.38)
Gender:Male	-	-	-0.02 (-0.32, 0.27)	-0.02 (-0.29, 0.26)
BMI:Obese	-	-	0.28 (-0.02, 0.57)	0.29 (6.3e-03, 0.56)
Comorbidity:Yes	-	-	0.22 (-0.03, 0.48)	0.10 (-0.17, 0.38)
Arm:Hybrid	-	-	0.49 (0.24, 0.74)	0.63 (0.38, 0.88)
Number of vaccines before Baseline	-	-	0.21 (0.05, 0.38)	0.21 (0.01, 0.41)
Infection/vaccination time to Baseline	-	-	0.001 (-0.0022, 0.0003)	-0.0009 (-0.0023, 0.0005)
**Omicron BA.5**				
Age:70+ years	0.97 (0.29, 3.23)	0.92 (0.17, 5.04)	0.14 (-0.12, 0.40)	0.05 (-0.17, 0.28)
Gender:Male	0.33 (0.11, 1.01)	0.24 (0.06, 0.93)	-0.006 (-0.28, 0.26)	-0.008 (-0.21, 0.20)
BMI:Obese	-	-	0.06 (-0.20, 0.33)	0.15 (-0.04, 0.35)
Comorbidity:Yes	0.61 (0.2, 1.91)	0.35 (0.07, 1.79)	0.08 (-0.15, 0.32)	0.11 (-0.10, 0.31)
Arm:Hybrid	7.3 (1.58, 33.64)	11.56 (2.26, 59.12)	1.03 (0.85, 1.20)	1.08 (0.89, 1.27)
Number of vaccination before Baseline	2.08 (1.08, 4.03)	2.87 (0.76, 10.86)	-0.11 (-0.27, 0.05)	0.03 (-0.11, 0.17)
Infection/vaccination time to Baseline	0.99 (0.99, 1)	1 (0.99, 1)	0.0012 (0, 0.0024)	-0.0004 (-0.0014, 0.0006)
**Omicron XBB.1.5**				
Age:70+ years	0.99 (0.5, 1.94)	0.98 (0.37, 2.59)	0.24 (0.01, 0.47)	0.18 (-0.06, 0.42)
Gender:Male	0.59 (0.3, 1.17)	0.57 (0.25, 1.35)	-0.07 (-0.32, 0.18)	-0.11 (-0.32, 0.11)
BMI:Obese	1.66 (0.78, 3.5)	2.15 (0.89, 5.22)	0.01 (-0.22, 0.25)	0.10 (-0.10, 0.30)
Comorbidity:Yes	0.91 (0.49, 1.7)	1.13 (0.48, 2.69)	0.13 (-0.08, 0.35)	0.08 (-0.13, 0.28)
Arm:Hybrid	12.54 (5.71, 27.52)	17.86 (7.29, 43.76)	0.70 (0.51, 0.89)	0.71 (0.50, 0.92)
Number of vaccination up to Baseline	0.86 (0.57, 1.31)	0.97 (0.49, 1.92)	-0.02 (-0.16, 0.12)	0.05 (-0.09, 0.19)
Infection/vaccination time to Baseline	1.0004 (0.9973, 1.0035)	1 (0.99, 1)	0.001 (-0.0001, 0.002)	0.0004 (-0.0006, 0.0014)
**Omicron EG.5**				
Age:70+ years	0.95 (0.49, 1.82)	0.88 (0.35, 2.23)	0.28 (0.05, 0.50)	0.11 (-0.13, 0.36)
Gender:Male	0.61 (0.32, 1.19)	0.59 (0.26, 1.36)	0.02 (-0.23, 0.27)	-0.007 (-0.23, 0.22)
BMI:Obese	1.2 (0.6, 2.38)	1.34 (0.58, 3.09)	0.16 (-0.07, 0.40)	0.20 (-0.006, 0.41)
Comorbidity:Yes	0.92 (0.51, 1.67)	1.24 (0.54, 2.84)	0.22 (0.01, 0.42)	0.11 (-0.11, 0.32)
Arm:Hybrid	11.59 (5.69, 23.63)	13.36 (6.13, 29.11)	0.58 (0.37, 0.80)	0.61 (0.38, 0.83)
Number of vaccines before Baseline	0.79 (0.53, 1.17)	1.07 (0.59, 1.94)	0.06 (-0.08, 0.21)	0.11 (-0.03, 0.25)
Infection/vaccination time to Baseline	1.002 (0.999, 1.005)	0.999 (0.995, 1.003)	0.0009 (-0.0002, 0.002)	0.0003 (-0.0007, 0.0013)

Table S3: Univariable/multivariable analysis to assess predictors of neutralizing antibody detectability and titers at baseline for Wuhan and the Omicron variants. OR- Odds ratio, adOR- adjusted Odds ratio, β- linear regression coefficient in univariable case, adβ- linear regression coefficient in multivariable case.

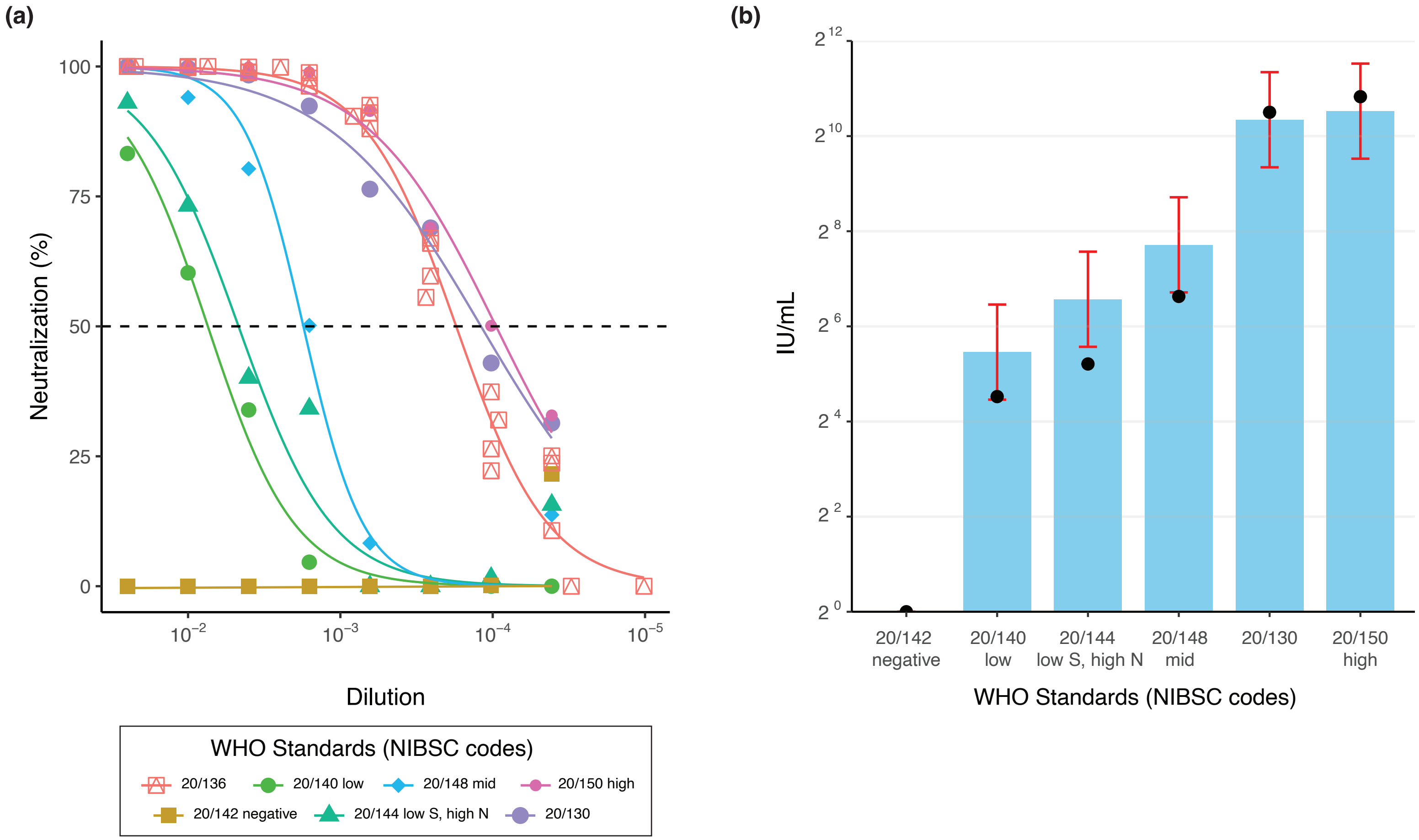

Figure S1. Neutralization assay calibration to the WHO International Standard (NIBSC code 20/136) and validation using the WHO International Reference Panel (NIBSC code 20/268) and a Research Reagent (NIBSC code 20/130). (a) Serial dilutions were performed for the first WHO International Standard for anti-SARS-CoV-2 immunoglobulin (n=4), and 6 WHO reference standards (n=1 each) and tested in a neutralization assay against pseudotyped-spike Wuhan (D614G) lentivirus. A dose-response curve was fitted for each WHO standard, and the dilution corresponding to 50% neutralization (ID50) (dashed line) was determined. (b) The first WHO International Standard (NIBSC code 20/136) is assigned an arbitrary value of 1000 IU/mL. In the neutralization assay (from (a)), the geometric mean of its 1/ID50 values is 5783. To convert 1/ID50 values to IU/mL for the other WHO standards, we divided their 1/ID50 by the ratio of 20/136's 1/ID50 value over its IU/mL value (5783/1000 = 5.783). The bar graph shows the geometric mean for each WHO standard, and the error bars represent a 0.5- to 2-fold range from the geometric mean. The black point shows the determined IU/mL for the indicated sample after conversion from its 1/ID50 value. For the 6 samples measured, 4 fall within the two-fold range of the WHO-reported values and two are slightly below this range.

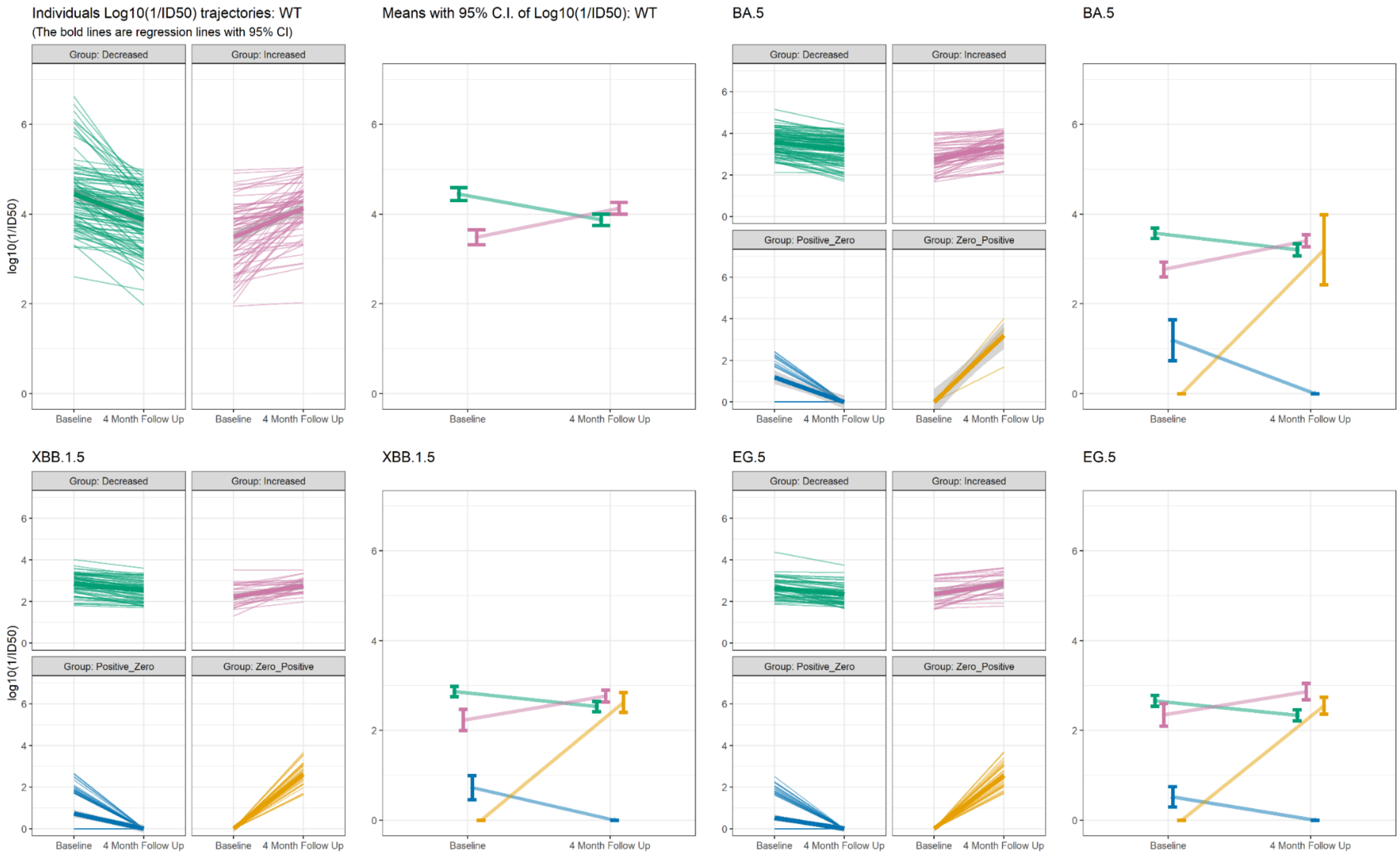

Figure S2: The trajectory patterns of neutralizing antibodies and mean changes observed at baseline and the 4-month follow-up for Wuhan (left 4 panels) and the Omicron variants. (right 4 panels).

Characteristics	Wuhan			Omicron BA.5				
	Decreased	Increased	P-value	Decreased	Increased	Positive-Zero	Zero-Positive	P-value
**Total (N)**	104	74	-	94	58	21	5	-
**Neutralizing antibody titer at baseline log10(1/ID50)**								
Mean (SD)	4.45 (0.74)	3.48 (0.73)	<.0001	3.57 (0.58)	2.77 (0.63)	1.19 (1.07)	0.00 (0.00)	<.0001
Median (IQR)	4.4 (3.9, 4.8)	3.6 (2.9, 4.0)	<.0001	3.6 (3.1, 4.0)	2.6 (2.3, 3.3)	1.7 (0.0, 2.2)	0.0 (0.0, 0.0)	<.0001
**4 month follow up log10(1/D50)**								
Mean (SD)	3.87 (0.64)	4.13 (0.58)	0.0071	3.20 (0.67)	3.40 (0.52)	0.00 (0.00)	3.20 (0.89)	<.0001
Median (IQR)	3.9 (3.4, 4.4)	4.2 (3.9, 4.5)	0.0064	3.4 (2.7, 3.7)	3.4 (3.1, 3.8)	0.0 (0.0, 0.0)	3.4 (3.3, 3.6)	<.0001
**Immunity status at baseline**								
Hybrid	57 (54.8%)	35 (47.3%)	0.323	67 (71.3%)	23 (39.7%)	2 (9.5%)	0 (0.0%)	<.0001
Vaccine	47 (45.2%)	39 (52.7%)		27 (28.7%)	35 (60.3%)	19 (90.5%)	5 (100.0%)	
**Age, n (%)**								
30-50 years	25 (24.0%)	27 (36.5%)	0.0719	27 (28.7%)	20 (34.5%)	4 (19.0%)	1 (20.0%)	0.5632
70+ years	79 (76.0%)	47 (63.5%)		67 (71.3%)	38 (65.5%)	17 (81.0%)	4 (80.0%)	
Mean (SD)	65.97 (14.50)	61.84 (16.23)	0.0763	64.15 (15.52)	62.69 (16.40)	68.71 (11.33)	65.60 (13.83)	0.4928
Median (IQR)	72 (69, 74)	71 (45, 74)	0.1353	71 (47, 74)	72 (46, 74)	72 (69, 74)	71 (70, 72)	0.6551
**Gender, n (%)**								
Female	78 (75.0%)	52 (70.3%)	0.4834	71 (75.5%)	44 (75.9%)	13 (61.9%)	2 (40.0%)	0.2015
Male	26 (25.0%)	22 (29.7%)		23 (24.5%)	14 (24.1%)	8 (38.1%)	3 (60.0%)	
**BMI, n (%)**								
Obesity (BMI ≥ 30)								
No	70 (67.3%)	63 (85.1%)	0.007	67 (71.3%)	44 (75.9%)	17 (81.0%)	5 (100.0%)	0.4312
Yes	34 (32.7%)	11 (14.9%)		27 (28.7%)	14 (24.1%)	4 (19.0%)	0 (0.0%)	
Mean (SD)	27.9 (5.6)	26.8 (6.6)	0.2581	27.8 (5.8)	27.6 (6.9)	26.1 (4.7)	25.1 (3.7)	0.5397
Median (IQR)	26.5 (23.7, 31.5)	25.0 (23.0, 28.0)	0.0649	26.0 (23.7, 30.7)	25.7 (23.0, 29.8)	25.1 (23.0, 27.4)	26.7 (25.8, 27.0)	0.5599
**Racial background, n (%)**								
White	95 (91.3%)	65 (87.8%)	0.4442	82 (87.2%)	54 (93.1%)	20 (95.2%)	4 (80.0%)	0.4597
Other	9 (8.7%)	9 (12.2%)		12 (12.8%)	4 (6.9%)	1 (4.8%)	1 (20.0%)	
**Smoking status, n (%)**								

Never	61 (58.7%)	42 (56.8%)	0.2083	55 (58.5%)	34 (58.6%)	10 (47.6%)	4 (80.0%)	0.8791
Previous	42 (40.4%)	28 (37.8%)		36 (38.3%)	23 (39.7%)	10 (47.6%)	1 (20.0%)	
Current	1 (1.0%)	4 (5.4%)		3 (3.2%)	1 (1.7%)	1 (4.8%)	0 (0.0%)	
**Comorbidities at baseline, diabetes or cardiovascular or respiratory or cancer, n (%)**								
No	44 (42.3%)	39 (52.7%)	0.1706	48 (51.1%)	27 (46.6%)	6 (28.6%)	2 (40.0%)	0.3102
Yes	60 (57.7%)	35 (47.3%)		46 (48.9%)	31 (53.4%)	15 (71.4%)	3 (60.0%)	
**Vaccination within 4 month follow up, n (%)**								
No	87 (83.7%)	41 (55.4%)	<.0001	78 (83.0%)	31 (53.4%)	17 (81.0%)	2 (40.0%)	0.0003
Yes	17 (16.3%)	33 (44.6%)		16 (17.0%)	27 (46.6%)	4 (19.0%)	3 (60.0%)	
**Breakthrough COVID-19 infection between baseline and 4-month follow up, n (%)**								
No	90 (86.5%)	56 (75.7%)	0.0629	87 (92.6%)	36 (62.1%)	21 (100.0%)	2 (40.0%)	<.0001
Yes	14 (13.5%)	18 (24.3%)		7 (7.4%)	22 (37.9%)	0 (0.0%)	3 (60.0%)	

Table S4a: Neutralizing antibody titers and predictors of the trajectory pattern groups for Wuhan and the Omicron BA.5 variants. n- number, BMI- body mass index, SD- standard deviation, IQR- interquartile range.

Characteristics	Omicron XBB.1.5					Omicron EG.5				
	Decreased	Increased	Positive-Zero	Zero-Positive	P-value	Decreased	Increased	Positive-Zero	Zero-Positive	P-value
**Total (N)**	68	30	53	27	-	55	31	60	32	-
**Neutralizing antibody titer at baseline log10(1/ID50)**										
Mean (SD)	2.87 (0.48)	2.23 (0.65)	0.73 (1.00)	0.00 (0.00)	<.0001	2.66 (0.46)	2.35 (0.71)	0.52 (0.88)	0.00 (0.00)	<.0001
Median (IQR)	2.9 (2.6, 3.2)	2.3 (1.8, 2.8)	0.0 (0.0, 1.8)	0.0 (0.0, 0.0)	<.0001	2.6 (2.3, 3.0)	2.4 (1.9, 3.0)	0.0 (0.0, 1.6)	0.0 (0.0, 0.0)	<.0001
**4 month follow up log10(1/D50)**										
Mean (SD)	2.53 (0.48)	2.76 (0.36)	0.00 (0.00)	2.62 (0.58)	<.0001	2.34 (0.48)	2.86 (0.50)	0.00 (0.00)	2.55 (0.56)	<.0001
Median (IQR)	2.6 (2.1, 2.9)	2.8 (2.5, 3.0)	0.0 (0.0, 0.0)	2.6 (2.2, 3.1)	<.0001	2.3 (1.9, 2.7)	2.9 (2.6, 3.3)	0.0 (0.0, 0.0)	2.5 (2.1, 3.0)	<.0001
**Immune status at baseline**										
Hybrid	59 (86.8%)	20 (66.7%)	11 (20.8%)	2 (7.4%)	<.0001	48 (87.3%)	24 (77.4%)	15 (25.0%)	5 (15.6%)	<.0001
Vaccine	9 (13.2%)	10 (33.3%)	42 (79.2%)	25 (92.6%)		7 (12.7%)	7 (22.6%)	45 (75.0%)	27 (84.4%)	
**Age, n (%)**										
30-50 year	18 (26.5%)	9 (30.0%)	17 (32.1%)	8 (29.6%)	0.9257	11 (20.0%)	12 (38.7%)	20 (33.3%)	9 (28.1%)	0.2488
70+ years	50 (73.5%)	21 (70.0%)	36 (67.9%)	19 (70.4%)		44 (80.0%)	19 (61.3%)	40 (66.7%)	23 (71.9%)	
Mean (SD)	65.31 (14.90)	63.33 (16.77)	63.89 (15.86)	63.33 (14.40)	0.9077	67.38 (14.23)	60.19 (16.31)	63.32 (15.96)	64.56 (14.57)	0.1957
Median (IQR)	72.0 (48.0, 75.0)	70.5 (45.0, 74.0)	71.0 (48.0, 74.0)	70.0 (49.0, 73.0)	0.7139	72.0 (70.0, 75.0)	70.0 (44.0, 73.0)	71.0 (46.5, 74.0)	70.5 (49.0, 74.0)	0.1024
**Gender, n (%)**										
Female	54 (79.4%)	23 (76.7%)	34 (64.2%)	19 (70.4%)	0.2808	44 (80.0%)	22 (71.0%)	40 (66.7%)	24 (75.0%)	0.4368
Male	14 (20.6%)	7 (23.3%)	19 (35.8%)	8 (29.6%)		11 (20.0%)	9 (29.0%)	20 (33.3%)	8 (25.0%)	
**BMI, n (%)**										
Obesity (BMI ≥ 30)										
No	52 (76.5%)	20 (66.7%)	39 (73.6%)	22 (81.5%)	0.6084	42 (76.4%)	22 (71.0%)	44 (73.3%)	25 (78.1%)	0.9039
Yes	16 (23.5%)	10 (33.3%)	14 (26.4%)	5 (18.5%)		13 (23.6%)	9 (29.0%)	16 (26.7%)	7 (21.9%)	
Mean (SD)	27.0 (5.1)	28.1 (7.8)	27.9 (6.3)	27.0 (5.7)	0.771	27.0 (6.1)	27.9 (6.1)	27.9 (6.2)	26.8 (5.8)	0.7512
Median (IQR)	25.5 (23.7, 29.8)	24.5 (22.8, 31.6)	26.5 (23.7, 30.0)	26.6 (23.3, 29.2)	0.9233	25.4 (23.5, 30.0)	25.8 (23.4, 31.4)	26.7 (23.7, 30.3)	25.3 (23.1, 29.5)	0.6629
**Racial background, n (%)**										

White	59 (86.8%)	28 (93.3%)	49 (92.5%)	24 (88.9%)	0.6743	49 (89.1%)	27 (87.1%)	54 (90.0%)	30 (93.8%)	0.8423
Other	9 (13.2%)	2 (6.7%)	4 (7.5%)	3 (11.1%)		6 (10.9%)	4 (12.9%)	6 (10.0%)	2 (6.3%)	
**Smoking status, n (%)**										
Never	40 (58.8%)	18 (60.0%)	28 (52.8%)	17 (63.0%)	0.7704	33 (60.0%)	19 (61.3%)	31 (51.7%)	20 (62.5%)	0.398
Previous	27 (39.7%)	11 (36.7%)	22 (41.5%)	10 (37.0%)		22 (40.0%)	10 (32.3%)	26 (43.3%)	12 (37.5%)	
Current	1 (1.5%)	1 (3.3%)	3 (5.7%)	0 (0.0%)		0 (0.0%)	2 (6.5%)	3 (5.0%)	0 (0.0%)	
**Comorbidities at baseline, diabetes or cardiovascular or respiratory or cancer, n (%)**										
No	30 (44.1%)	14 (46.7%)	25 (47.2%)	14 (51.9%)	0.9245	23 (41.8%)	16 (51.6%)	30 (50.0%)	14 (43.8%)	0.7527
Yes	38 (55.9%)	16 (53.3%)	28 (52.8%)	13 (48.1%)		32 (58.2%)	15 (48.4%)	30 (50.0%)	18 (56.3%)	
**Vaccination within 4 month follow up, n (%)**										
No	58 (85.3%)	13 (43.3%)	41 (77.4%)	16 (59.3%)	0.0001	48 (87.3%)	13 (41.9%)	46 (76.7%)	21 (65.6%)	<.0001
Yes	10 (14.7%)	17 (56.7%)	12 (22.6%)	11 (40.7%)		7 (12.7%)	18 (58.1%)	14 (23.3%)	11 (34.4%)	
**Breakthrough COVID-19 infection between baseline and 4-month follow up, n (%)**										
No	64 (94.1%)	22 (73.3%)	52 (98.1%)	8 (29.6%)	<.0001	52 (94.5%)	24 (77.4%)	59 (98.3%)	11 (34.4%)	<.0001
Yes	4 (5.9%)	8 (26.7%)	1 (1.9%)	19 (70.4%)		3 (5.5%)	7 (22.6%)	1 (1.7%)	21 (65.6%)	

Table S4b: Neutralizing antibody titers and predictors of the trajectory pattern groups for the Omicron XBB.1.5 and EG.5 variants. n- number, BMI- body mass index, SD- standard deviation, IQR- interquartile range.

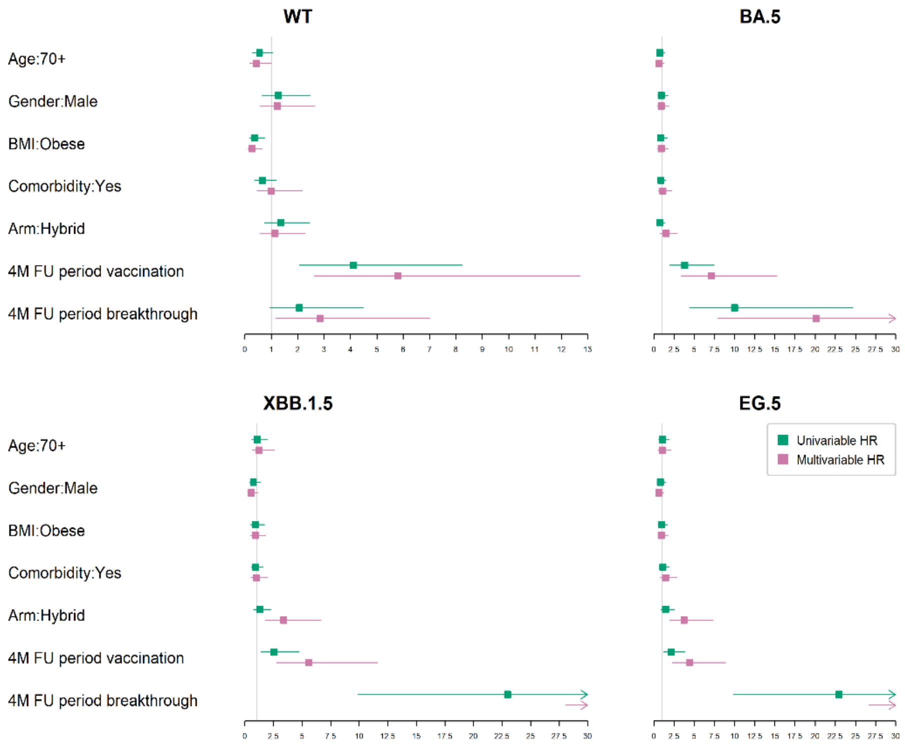

Figure S3: Predictors of neutralizing antibody titers level change during 4-month follow-up for Wuhan (WT), (upper left). Omicron BA/5 (upper right, Omicron XBB1.5 (lower left) and Omicron EG.5 (lower right).

	Short term (4 months) follow up			Long term follow up (10 months, up to 22 September 2023)		
	Non-infected	Infected	P-value	Non-infected	Infected	P-value
Total, N	146	32	-	110	68	-
Age groups, n(%)						
30-50 years	43 (29.5%)	9 (28.1%)	0.8812	33 (30.0%)	19 (27.9%)	0.7691
70+ years	103 (70.5%)	23 (71.9%)		77 (70.0%)	49 (72.1%)	
Gender, n(%)						
Female	107 (73.3%)	23 (71.9%)	0.8705	79 (71.8%)	51 (75.0%)	0.6421
Male	39 (26.7%)	9 (28.1%)		31 (28.2%)	17 (25.0%)	
Obesity (BMI ≥ 30)						
No	107 (73.3%)	26 (81.3%)	0.3479	82 (74.5%)	51 (75.0%)	0.9459
Yes	39 (26.7%)	6 (18.8%)		28 (25.5%)	17 (25.0%)	
Comorbidities: diabetes or cardiovascular or respiratory or cancer, n(%)						
No	68 (46.6%)	15 (46.9%)	0.9755	55 (50.0%)	28 (41.2%)	0.2516
Ye	78 (53.4%)	17 (53.1%)		55 (50.0%)	40 (58.8%)	
Immune status at baseline, n(%)						
Hybrid	84 (57.5%)	8 (25.0%)	0.0009	61 (55.5%)	31 (45.6%)	0.2006
Vaccine	62 (42.5%)	24 (75.0%)		49 (44.5%)	37 (54.4%)	
BA.5 antibody value at baseline (log10 1/ID50)						
Mean (SD)	3.01 (1.12)	2.54 (1.06)	0.0309	2.93 (1.21)	2.93 (0.98)	0.9806
Median (IQR)	3.2 (2.6, 3.8)	2.6 (2.1, 3.1)	0.0034	3.2 (2.4, 3.8)	3.0 (2.5, 3.6)	0.3086
Time to breakthrough from baseline visit (months)						
Mean (SD)	-	70.66 (41.99)	-	-	126.04 (88.17)	-
Median (IQR)	-	62.5 (38.0, 112.0)	-	-	127.5 (62.5, 149.5)	-
Vaccination during 4 month follow up period						
No	101 (69.2%)	27 (84.4%)	0.0832	56 (50.9%)	50 (73.5%)	0.0028
Yes	45 (30.8%)	5 (15.6%)		54 (49.1%)	18 (26.5%)	

Table S5: Distribution of predictors of breakthrough COVID-19 infection during short/long term. n- number, BMI- body mass index, SD- standard deviation, IQR- interquartile range.
